# Physical activity, energy requirements, and adequacy of dietary intakes of older persons in a rural Filipino community

**DOI:** 10.1186/1475-2891-8-19

**Published:** 2009-05-04

**Authors:** Maria Grace D Risonar, Pura Rayco-Solon, Judy D Ribaya-Mercado, Juan Antonio A Solon, Aegina B Cabalda, Lorena W Tengco, Florentino S Solon

**Affiliations:** 1 Nutrition Center of the Philippines, Taguig City, Philippines; 2 Human Nutrition Research Center at Tufts University, Boston, Massachusetts, USA; 3 University of the Philippines, Ermita District, Manila, Philippines

## Abstract

**Background:**

Aging is a process associated with physiological changes such as in body composition, energy expenditure and physical activity. Data on energy and nutrient intake adequacy among elderly is important for disease prevention, health maintenance and program development.

**Methods:**

This descriptive cross-sectional study was designed to determine the energy requirements and adequacy of energy and nutrient intakes of older persons living in private households in a rural Filipino community. Study participants were generally-healthy, ambulatory, and community living elderly aged 60–100 y (n = 98), 88 of whom provided dietary information in three nonconsecutive 24-hour food-recall interviews.

**Results:**

There was a decrease in both physical activity and food intake with increasing years. Based on total energy expenditure and controlling for age, gender and socio-economic status, the average energy requirement for near-old (≥ 60 to < 65 y) males was 2074 kcal/d, with lower requirements, 1919 and 1699 kcal/d for the young-old (≥ 65 to < 75 y) and the old-old (≥ 75 y), respectively. Among females, the average energy requirements for the 3 age categories were 1712, 1662, and 1398 kcal/d, respectively. Actual energy intakes, however, were only ~65% adequate for all subjects as compared to energy expenditure. Protein, fat, and micronutrients (vitamins A and C, thiamin, riboflavin, iron and calcium) intakes were only ~24–51% of the recommended daily intake. Among this population, there was a weight decrease of 100 g (p = 0.012) and a BMI decrease of 0.04 kg/m^2 ^(p = 0.003) for every 1% decrease in total caloric intake as percentage of the total energy expenditure requirements.

**Conclusion:**

These community living elderly suffer from lack of both macronutrient intake as compared with energy requirements, and micronutrient intake as compared with the standard dietary recommendations. Their energy intakes are ~65% of the amounts required based on their total energy expenditure. Though their intakes decrease with increasing age, so do their energy expenditure, making their relative insufficiency of food intake stable with age.

## Background

The world population is now experiencing population aging as evidenced by the much more rapid growth of the sector of persons ≥ 60 y compared to that of the general population [1]. Life expectancy globally is expected to increase by 11 years, from 65 in 1995–2000 to 76 in 2045–2050 with 597 million people ≥ 60 y living in developing countries [2]. A 2000 national census showed that there are about 4.6 million older persons living in the Philippines [3].

The increasing population of older persons may lead to an increase in the number of people at high risk of disability and morbidity. Older persons have special health needs and nutritional requirements arising from long-standing dietary habits, a lifetime of different disease encounters and changes in body structure and metabolism. In view of this changing trend, the older age group will become a globally significant health, social and economic policy concern of governments. The development of sound, affordable and sustainable policies and effective community-based programs is necessary to respond to their needs.

There are limited data on the nutrient requirements of older persons that could be used as a basis for health policy. Partly, the dearth of data may be due to the inherent difficulty of collecting dietary data. Another factor may be the presence of different groups of older persons (community living [4-6], homebound [7], or institutionalized [8,9]; healthy [10,11], frail [12-15] or sick [16,17]), with the resulting difficulty in generalization of recommendations [18].

In addition, assessment of the nutritional situation often does not adjust for energy expenditure. Testing hypothesis regarding the adequacy of energy and nutrient intakes in the young- and old-old compared to the near-old emphasizes the changing requirements through age in the context of decreasing energy expenditure [19,20].

This cross-sectional study was designed to determine the adequacy of energy and nutrient intakes compared to the energy requirements of community living older persons in private households in rural Philippines, with emphasis on how this picture changes as the person ages and becomes less active.

## Methods

### Study site and participants

This study was carried out among generally-healthy, ambulatory, and community living Filipino older persons (≥ 60 y old) in the rural villages of Palsara and Malabanan in the municipality of Balete, Batangas province, Philippines. The poverty prevalence in Balete is 50.1% (national prevalence is 27%) [21]. They had low socio-economic status and a high prevalence of malnutrition among schoolchildren, where 48% had low vitamin A intake and prevalence of anemia was 55% [5]. The villages of Palsara and Malabanan were chosen as study sites because they have a large elderly population as recorded in the local Department of Social Welfare and Development (DSWD). The DSWD list of 179 older persons from these villages was validated through house-to-house visits by field staff with the help of the local health workers. There were only 179 older persons in the community. Seventy-nine persons were excluded because they were non-ambulatory (3 persons); had difficulty of breathing from chronic obstructive pulmonary disease or asthma (15 persons); had acute illnesses at the time of the interview such as fever (7), diarrhea (2), cough (6), difficulty in urination (10), weight loss (7), active tuberculosis (5) and malaria (8); or had history of past major illnesses such as cardiovascular diseases (9), stroke (7). A total of 100 older persons with no history of major illnesses and who were ambulatory were invited to participate in the study. They were living in private residences and were not home-bound or institutionalized; they were fully independent in performing daily tasks. These older persons were interviewed to determine their age, medical history and dietary habits. There were 98 older persons who consented to take part in the study, and 88 provided complete dietary information. They were categorized according to age: ≥ 60 to < 65 y as near-old group, ≥ 65 to < 75 y as young-old group, and ≥ 75 y as old-old group [22]. Those who took part in the study were not significantly different from those who were excluded from the study in terms of age (t-test p = 0.383) and gender (chi-square p = 0.926).

### Data collection

Nurses and nutritionists conducted house-to-house interviews to obtain socio-economic demographic profiles and medical histories. Information such as the participant's age, educational attainment, family size, household composition, occupation and average monthly income were gathered. A medical history form was used to gather information on the participant's medical history such as past and present illnesses, use of medications and nutritional supplements, and smoking and alcohol drinking habits. Smoking was defined as currently smoking at least one cigarette per week. Drinking was defined as intake of at least one unit per day (one bottle of beer or one glass of rum or local liquor distilled from grains).

A trained physician conducted a physical examination for clinical signs of illness and validated the information in the medical history form. Weight was measured in light clothing using a platform scale (Detecto, Missouri, USA) and height was measured in upright standing position without shoes using a standard portable wooden height measuring board (UNICEF model). Weight was recorded to the nearest 0.1 kg and height to the nearest 0.1 cm. Weights and heights were used to calculate body mass index (BMI = kg/m^2^). Medical technologists collected 5 ml of blood samples by venipuncture after an 8-hour fast for blood sugar and cholesterol determinations. These blood indices were reported more fully in an earlier publication [7].

Registered dietitians conducted three non-consecutive 24-hour dietary recalls (two weekdays and one weekend). Food models and household measuring tools were used by dietitians to aid the subjects in estimating their food intakes. Portion sizes were estimated using measuring cups and spoons and food models. Dietary intakes of fat, protein, carbohydrates, and the micronutrients were assessed by using the Philippine Food Composition Tables [23]. The results from the three dietary recalls were averaged.

The assessment of physical activity level was done by observation. The field staff followed the subjects during their routine daily activities in the span of 2 months, averaging about 2 to 3 days of observation per subject in the course of data collection and fieldwork (e.g., dietary recall, medical history and socio-economic interviews, collection of samples, counseling on prescribed medications). During a single observation period, the field staff stayed with the subjects from 4 to 6 hours at a time. The subjects' activities were variously observed in the morning, afternoon and evening. In this way the field staff were able to collect data on light to moderate intensity activities such as leisure activities, sitting, eating, and commuting which would have been more difficult to recall accurately. The list of physical activities and their level of intensity expressed as multiples of the basal metabolic rate [24] is shown in Table 1.

**Table 1:** Physical activity level (PAL) energy cost expressed as multiples of the Basal Metabolic Rate (WHO, 1985) with definition and examples.

**Sedentary to moderate activities (PAL Energy Cost 1.40 to 1.99)**		
Sleeping*	8 hours of sleep	1.0
Commuting	Commuting to/from work or farm on the bus or jeepney	1.2
Leisure activities	Light leisure activities, watching television, chatting	1.4
Sitting	Sitting, office work, selling produce, tending shop	1.5
Eating	Eating	1.5
**Vigorous activities (PAL Energy Cost 2.0 to 2.4)**		
Driving	Driving car or jeepney to/from work or farm	2.0
Cooking	Cooking	2.1
Standing	Standing carrying light loads, waiting on tables, arranging merchandise	2.2
Personal care	Personal care, dressing, showering	2.3
Chores	Non-mechanized domestic chores, sweeping, washing clothes, doing dishes by hand	2.3
**Hard work (PAL Energy Cost >2.5)**		
Household work	General household work	2.8
Walking	Walking at varying paces without a load	3.2
Agricultural work	Non-mechanical agricultural work, planting, weeding, gathering	4.1
Carrying load	Collecting water or wood	4.4

*8 hours of sleep is equivalent to 1.0 energy cost

Mean micronutrient intakes were computed as percentages of the recommended intake based on the Philippine Recommended Energy and Nutrient Intakes [25]; average macronutrient intakes were calculated as percentages based on the basal metabolic rate (BMR) predicted by the desirable body weight (DBW), using the formulas in Table 2. BMR was computed as 1 kcal per kg of DBW per hour. DBW (in kg) was computed by subtracting the factor of 100 from the value of height (in cm) and taking off 10% which is applicable to Filipino stature [26]. Estimating energy requirements for this group of older persons was computed by the caloric allowance per kg DBW based on their physical activity levels (PAL).

**Table 2:** Formulas for calculating energy requirements, macronutrient allowances and adequacy of intake of macronutrients in Filipino elders.

**Reference**		**Formulas**
Desired Body Weight (kg)^1^	DBW	(height in cm - 100) × 0.9
Basal Metabolic Requirement (kcal)	BMR	1 kcal/kg × DBW in kg × hours
Caloric allowance based on physical activity level	PAL	
Sedentary or light activity		35 kcal/kg × DBW in kg × hours in light activities
Active or moderately active		40 kcal/kg × DBW in kg × hours in moderately active activities
Vigorous or vigorously active		45 kcal/kg × DBW in kg × hours in vigorous activities
Specific Dynamic Action of food (kcal)	SDA	10% of (BMR + PAL)
Total Energy Requirement (kcal/day)	TER	BMR + PAL + SDA
Change in Energy Requirements based on age	CER	
≥ 60 to <70 y		10% decrease
≥ 70 y		20% decrease
Age-adjusted Total Energy		
Requirement (kcal/day)	ATER	TER - (TER × CER)
Macronutrient Allowance	Allowance	
Energy (kcal)		ATER in kcal
Protein (g)		(ATER in kcal × 0.60)/4 g per calorie
Fat (g)		(ATER in kcal × 0.25)/9 g per calorie
Carbohydrates (g)		(ATER in kcal × 0.15)/4 g per calorie
Adequacy of intake of macronutrients (%)	Adequacy	
Energy		(Total caloric intake/ATER) × 100
Protein		(Protein intake/Protein allowance) × 100
Fat		(Fat intake/Fat allowance) × 100
Carbohydrates		(Carbohydrate intake/Carbohydrate allowance) × 100
Mean Physical Activity Level	MPAL	(Hours spent per activity × Energy cost of activity)/24 hours

^1^Based on Querubin and Panlasigui (1994)

Socio-economic status was a score (0, 1 or 2) constructed from a matrix of toilet facilities and availability of drinking water. A socio-economic score (SES) of 1 was given to participants who either had a private water-sealed toilet or had a private source of drinking water (well, piped-in or electric pump). Those with a SES of 0 had neither and those with a SES of 2 had both of these. There was a significant correlation between this socio-economic score construct and the reported total monthly income of the household (Spearman's ⟩ = 0.327, p = 0.001).

### Data analysis

Data were encoded using FoxPro version 2.6 (Microsoft Corporation, Redmond, WA) and analyzed using EpiInfo version 6.04d (Centers for Disease Control and Prevention, USA and World Health Organization, Geneva, Switzerland) and Stata (Stata Corporation, College State, TX, USA). Means were computed for quantitative variables while frequency distribution tables were generated for categorical variables. Pearson's chi-square test, or Fisher's Exact test were used to analyze data in Table 3 regarding characteristics of study participants. Mean and standard deviation (SD) data categorized by age and sex were presented in Tables 4 and 5, showing the total energy requirements (Table 4) and the anthropometric measurements of study participants (Table 5). Simple linear regression analysis was used to analyze difference across different age groups for Tables 4 and 5.

**Table 3:** Characteristics of the study participants.

	**Age group**			P-value*
	**Near-old (N = 40)**	**Young-old (N = 32)**	**Old-old (N = 26)**	
Males, %	45.0	37.5	53.8	0.461
Live with others, %	97.5	100.0	92.3	0.231
Socio-economic score (SES)3				
0, %	23.1	12.9	12.0	0.606
1, %	25.6	29.0	20.0	
2, %	51.3	58.1	68.0	
Mean physical activity level (MPAL)4				
Light (MPAL >1.4 to <1.7), %	0.0	3.1	46.1	<0.001**
Moderately active (MPAL >1.7 to <2.0), %	12.5	65.6	38.5	
Vigorous (MPAL >2.0 to 2.4), %	87.5	31.3	15.4	
Smoker, %	45.0	34.4	50.0	0.459
Body Mass Index (BMI)5				
				
Chronic energy deficient (BMI <18.5), %	22.5	37.5	47.8	0.168**
Normal (BMI >18.5 to <25), %	55.0	56.2	47.8	
Overweight (BMI >25 to <30), %	20.0	6.3	4.4	
Obese (BMI >30), %	2.5	0.0	0.0	
With soil-transmitted helminths, %	54.1	56.7	64.0	0.733
With dental caries, %	40.0	41.9	38.5	0.965
With elevated cholesterol level, %	35.9	18.8	12.0	0.064
With goiter, %	12.5	21.9	7.7	0.282
With pulmonary tuberculosis, %	5.0	16.1	19.2	0.011

* Pearson's chi-square p-value

** Fisher's exact test p-value

**Table 4:** Total energy requirements (kcal/day) based on basal metabolic rate, specific dynamic action of food, and activity levels of the subjects, by age group. Simple linear regression p-values are shown across age groups.

	**Age group**						**P-value**
	**Near-old**		**Young-old**		**Old-old**		
	**Mean**	**SD**	**Mean**	**SD**	**Mean**	**SD**	
Males	2074	199	1919	241	1699	149	<0.001
Females	1712	149	1662	170	1398	131	<0.001
							
All	1875	250	1759	233	1555	206	<0.001

**Table 5:** Anthropometry measurements of study participants, by gender and age group. Simple linear regression p-values are shown across age groups.

	**Age group**						**P-value**
	**Near-old**		**Young-old**		**Old-old**		
	**Mean**	**SD**	**Mean**	**SD**	**Mean**	**SD**	
**Weight (kg)**							
Males	54.0	11.1	53.2	8.2	47.3	6.0	0.037
Females	50.3	10.7	43.4	7.3	41.5	7.5	0.004
							
All	52.0	10.9	47.1	8.9	44.6	7.2	0.004
**Height (cm)**							
Males	160.3	5.9	159.2	5.9	157.5	3.6	0.168
Females	150.0	4.6	151.2	4.1	148.4	4.3	0.479
							
All	154.7	7.3	154.2	6.2	153.2	6.1	0.394
**BMI (kg/m^2^)**							
Males	20.9	3.6	21.1	3.7	19.1	2.7	0.188
Females	22.3	4.0	19.0	3.2	18.6	3.3	0.003
							
All	21.7	3.8	19.8	3.5	18.9	3.0	0.006

**Figure 1:**
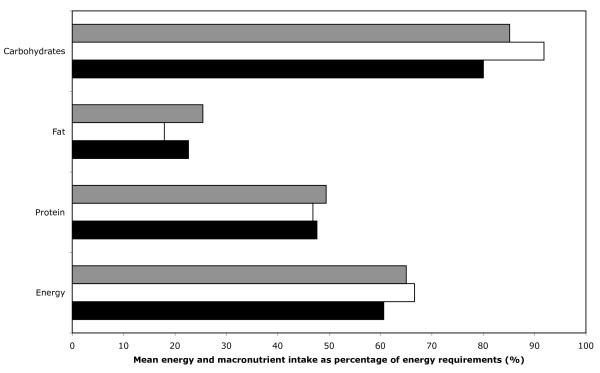
**Mean energy and macronutrient intake as percentage of the total energy requirements by age group, calculated from multiple linear regression analysis controlling for gender and SES**. Gray bar, near-old; white bar, young-old; black bar, old-old age group.

Multiple linear regression was used to analyze the data shown in Figures 1 (mean energy and macronutrient intake as percentage of the total energy requirements) and 2 (mean micronutrient intake as percentage of the recommended daily intakes). The co-variables included in these multiple linear regression analyses are age group, gender and SES. Multiple linear regression equations were also done for energy requirements with age group, gender and SES as co-variables. Anthropometric measurements were also analyzed using multiple linear regression with age group, gender, SES and the energy intakes as a percentage of the total energy expenditure as co-variables.

Because there were cases in which more than one subject lived in the same household, a multiple linear regression mixed effects model was used with the household number as a random effects parameter and the explanatory variables as fixed effects parameters when controlling for several variables at the same time. For all the tests, a p-value < 0.05 was considered to be statistically significant.

### Ethical approval

Approval to conduct the study was obtained from the Tufts University-New England Medical Center Human Investigation Review Committee and the National Ethics Committee of the Philippine Council for Health Research and Development.

## Results

A description of the subjects is shown in Table 3. Their mean age was 69 y, with a range of 60–100 y. In total, 40.8% of the participants were near-old, 32.7% were young-old, and 26.5% were old-old. No difference was noted in gender distribution per age group (p = 0.461). The 98 subjects belonged to 81 households, with 17 households housing two study participants each.

Their mean monthly household incomes were below the poverty threshold [4] and 42.1% did not have a private water-sealed toilet or a private source of drinking water, or both, in their homes (SES was either 1 or 0). Significantly more near-old subjects than either young-old or old-old (p < 0.001) had a mean physical activity level of ≥ 2.0 (vigorous activity). Their typical diet consisted mostly of rice, fish and vegetables. Chronic energy deficiency (defined as BMI of less than 18.5 kg/m^2^) was 22.5%, 37.3% and 47.8% among the near-old, young-old and the old-old respectively.

The average total energy requirements as calculated based on the basal metabolic rate, specific dynamic action of food, activity levels of the subjects and the analysis using simple linear regression are shown in Table 4. The table shows that the average total energy requirements decrease significantly with increasing age. Additional analysis using multiple regression analysis controlling for age, gender and socio-economic status showed that near-old males needed an average of 2074 kcal/d (95% confidence interval (CI): 1939 to 2140 kcal/d), young-old males needed 78 calories less (95% CI: -162 to 7 kcal/d; p = 0.07), and the old-old males needed 350 calories less (95% CI: -255 to -444 kcal/d; p < 0.001). Among females, the same trend for decreasing requirements was seen with increasing age. The total energy requirement was not significantly associated with socio-economic status (data not shown).

Figure 1 (and Additional file 1) shows the mean energy and macronutrient intakes of subjects as a percentage of their total energy requirements based on desired body weight, physical activity and age using the formulas given in Table 2. The intakes of the subjects for all the macronutrients had an average percentage of only 64.8% for energy intake, and 47.9%, 28.3%, and 84.1% for intakes of protein, fat and carbohydrates, respectively. The findings were not significantly different by age, sex or SES using the multiple linear regression analysis.

**Figure 2:**
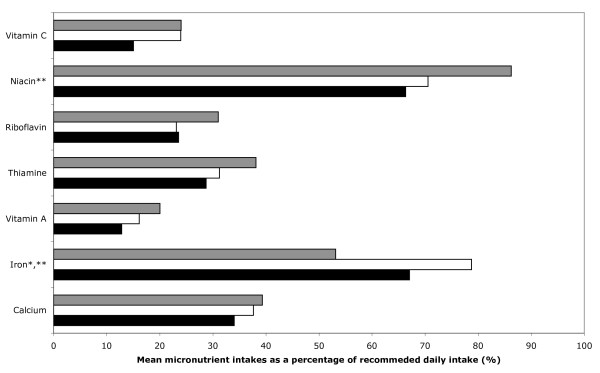
**Mean micronutrient intake as percentage of the recommended daily intakes by age group, calculated from multiple linear regression analysis controlling for gender and SES**. Gray bar, near-old; white bar, young-old; black bar, old-old age group. * P < 0.05 for the test evaluating the null hypothesis that the percentage among the near-old and the young-old are equal. ** P < 0.05 for the test evaluating the null hypothesis that the percentage among the near-old and the old-old are equal.

Figure 2 (and Additional file 2) shows the micronutrient intakes of the subjects as a percentage of the Philippine Recommended Energy and Nutrient Intakes [10]. All micronutrient intakes were had average percentages below 100%. For all subjects, the intakes of vitamin C, vitamin A, and calcium were about 24%, 27%, and 31% of the recommended intakes, respectively; those of thiamin, riboflavin, iron, and niacin were about 34%, 35%, 51%, and 77%, respectively. The percent intake of niacin was significantly less among the old-old compared with the near-old (p = 0.040). The iron intakes of the young-old and the old-old were significantly better than those of the near-old (young-old vs near old: multiple logistic regression coefficient = 25.6 mg, 95% CI = 13.1 to 38.0, p < 0.001; old-old vs near old: coef = 13.9 mg, 95% CI = 0.4 to 27.4, p = 0.44) but still did not reach 100% of recommended levels. There were no significant differences in intake percentage for riboflavin, thiamin, calcium, vitamin A and vitamin C among age groups.

Mean anthropometry measurements are shown in Table 5. Simple linear regression p-values shown in the table indicate that weight significantly decreases with older age in both genders and BMI significantly decreases with age among females. Additional analysis using multiple regression analysis controlling for age, gender, SES and energy intake as a percentage of the total expenditure showed progressively lower weight and body mass index (BMI) with increasing age, particularly among females. A weight decrease of 100 g (95% CI: 22 to 180; p = 0.01) and a BMI decrease of 0.04 kg/m^2 ^(95% CI: 0.02 to 0.07; p = 0.003) were observed for every 1% decrease in total caloric intake as a percentage of the total energy expenditure requirements. Height was not associated with energy intake percentage.

## Discussion

The main concern of geriatric nutrition is the conservation of good health and prevention of degenerative diseases [27]. Ideally, people should reach old age with sound eating habits in order to maintain adequate nutritional status.

This study provides energy and nutrient intake data from older persons independently living in private households in a rural community in the Philippines. For all subjects, intakes of carbohydrates, protein, and fat provided ~78%, 11%, and 11% of the total energy intake, respectively. While the actual food intake of the old-old were less than those of the near- and young-old, because of the decreasing energy requirements with increasing age, the relative intake of macronutrients as a percentage of energy expenditure was not significantly different for the different age groups. The percentages were not equally distributed among the macronutrients, however, with a much lower relative percentage in fat and protein intakes compared to carbohydrate intake.

These results are similar to findings from other recent studies among community-dwelling, community living elderly people in both developing and developed countries [5,6,15,17,28,29] wherein there is a need for older persons to achieve adequate nutrient intakes. These findings underline the need for a two-pronged approach for nutritional management of older people: (a) timely nutritional assessment, and (b) appropriate nutritional counseling [30-36]. Part of this intervention is to identify possible barriers to proper nutrition [37].

Many age-related changes that might influence dietary and health requirements occur continually throughout the life cycle. Micronutrient deficiencies have been shown to be common in elderly people due to a reduced food intake resulting from dietary restrictions and a lack of variety in the foods they eat. They are particularly vulnerable to dietary restrictions due to social and psychological factors, long standing dietary habits, physiological changes associated with aging [6] and oral problems such as impaired dentition, fewer natural teeth and dental caries [38-41]. Dental caries was a prevalent condition seen among our population of elderly.

More than a third of the participants were chronic energy deficient. Low food intake with subsequent low body weight, weakened immune system at old age and poor access to health facilities and services may further increase their risk for many nutrition-related acute or chronic illnesses.

Five of the 7 micronutrients with dietary intake data (vitamin A vitamin C, calcium, thiamin, riboflavin) were approximately 1/3 of the recommended nutrient intake for Filipino elderly. Iron (51%) and niacin (77%) were the exceptions. The dietary intake was particularly low for vitamins A and C. The percentage niacin intake decreased with increasing age. Iron intake as a percentage of the recommendations had significant variability with age, increasing among the young-old and the old-old compared with the near-old. This population also had data on vitamin A status [42]. Total body vitamin A measured by deuterated-retinol-dilution method, but not serum retinol was correlated with dietary vitamin A intake [42].

Studies conducted in neighboring Asian countries show that mean intakes among populations of older persons often fall below the official dietary recommendations. Among 350 elderly Malays [20] the mean intakes of energy and all nutrients were below the recommended allowances and among 240 community living Indonesian elderly [43] the median energy intake was below the assessed requirement while iron, thiamin and folate intakes were below the recommended values. It has been suggested that increasing both the protein and iron intakes together may be an efficient way of adding value to the diets of elders [44].

Financial status may limit one's dietary choices. Though most of the study participants were still economically active, their mean monthly incomes were below the national poverty threshold for rural areas [43]. In this population, however, socio-economic status did not show a significant association with dietary intake except for vitamin A intake which was higher among those in the highest SES bracket. There may be other confounding variables responsible for this association such as the educational level of the subjects and/or their housemates. Likewise, there might not have been enough variability to see an effect on the subjects' intakes.

The cross-sectional nature of data collection allowed for associations to be identified, but causality cannot be established. Furthermore, because our study participants were community living older persons who were generally healthy, the results of this study may not be directly applicable to institutionalized or home-based elderly even though they are consistent with other study results conducted among this age group [6,45-47]. The study area was rural and one of low socio-economic status, thus, the results might not be generalizable to urban-dwelling elders with higher socio-economic status. In addition, as in inherent to the use of the 24-hour food recalls, problems of recollection and measurements of portions may have been a factor in the data collection.

## Conclusion

The study results show that the community living elderly participants in this study had energy intakes that were about 65% of the amounts required based on their total energy expenditure. Their nutritional and health vulnerability, together with a myriad of age-related processes such as body structure changes, and physiological and immune function alterations make this sector of the population at high risk for malnutrition-related diseases. Also their growing population makes them a significant social and economic concern of governments.

Attempts to provide the elderly with adequate nutrition encounter many practical problems. First, their nutritional requirements are not well defined. Second, the process of aging also affects other nutrient needs. The effect of aging on nutrient requirements cannot be easily quantified. There are changes noted in studies that showed a decrease in energy requirement with advancing age [48], while other studies suggest that requirements for other essential nutrients may in fact increase in later life [49] such as protein requirements per kg of body weight and certain vitamins such as vitamins A and B_12 _that may be affected by chronic conditions. Thus, there is a need to review current recommended daily nutrient allowances which could be used as basis for the development of guidelines to address the nutritional needs of the growing elderly population.

Programs for this group may be geared towards (a) screening for and assessing nutritional problems, and (b) nutritional counseling, health promotion and disease prevention. Health care and support services should be made accessible and education and awareness on health and nutrition should be promoted not only among the older persons but also among younger adults. Efforts should be made in educating the public on the conditions associated with aging including effective elderly care. The importance of micronutrients in the promotion of health and prevention of later-life disorders should also receive considerable attention in diet-disease national programs.

## Competing interests

The authors declare that they have no competing interests.

## Authors' contributions

MGDR contributed to the acquisition of data, interpretation of data, and preparation of the manuscript. PRS analyzed and interpreted the data, and prepared the manuscript. JDRM and JAAS provided significant advice with respect to the analysis and interpretation of the data and critically reviewed the manuscript. AAB and LWT contributed to the collection of the data and review of the manuscript. FSS contributed to the design of the study and critically reviewed the manuscript.

## Supplementary Material

Additional file 1 **Energy and macronutrient intakes of the study participants, by age and gender**. Significant differences in the intake of energy, protein and carbohydrates (but not fat intake) with increasing age was seen using multiple logistic regression analysis, controlling for age, gender and SES. Click here for file

Additional file 2 **Micronutrient intakes of the study participants, by age, gender and SES score**. Intakes of iron, niacin, riboflavin, thiamin, calcium, vitamin A and vitamin C were not significantly different by age, sex or socio-economic status, except for vitamin A intake which was significantly higher for those with the highest SES. Click here for file
