# Supplementary material for: Physical activity, energy requirements, and adequacy of dietary intakes of older persons in a rural Filipino community
Source: Nutr J. 2009 May 4;8:19. doi: 10.1186/1475-2891-8-19 (PMC2689250; doi:10.1186/1475-2891-8-19)
Supplement: Additional file 1 — Energy and macronutrient intakes of the study participants, by age and gender. Significant differences in the intake of energy, protein and carbohydrates (but not fat intake) with increasing age was seen using multiple logistic regression analysis, controlling for age, gender and SES. [file 1475-2891-8-19-S1.doc]

Additional file 1

Significant differences in the intake of energy, protein and carbohydrates (but not fat intake) with increasing age was seen using multiple logistic regression analysis, controlling for age, gender and SES. The difference in protein intake between genders was significant, with females consuming 5 g less than males (p = 0.03). The contributions of carbohydrate, fat and protein to the total energy intake of males were 78.3%, 9.9%, and 11.5%, respectively; the contributions to the total energy intake of females were 77.4%, 11.6%, and 10.6%, respectively.

|  | **Energy (kcal)** | | | **Protein (g)** | | | **Fat (g)** | | | **Carbohydrates (g)** | | |
| --- | --- | --- | --- | --- | --- | --- | --- | --- | --- | --- | --- | --- |
| Age group |  |  |  |  |  |  |  |  |  |  |  |  |
| Near-old | 1,200 | + | 416 | 34 | + | 13 | 16 | + | 10 | 229 | + | 88 |
| Young-old | 1,195 | + | 456 | 31 | + | 14 | 12 | + | 6 | 240 | + | 100 |
| Old-old | 922 | + | 302 | 27 | + | 11 | 11 | + | 11 | 178 | + | 64 |
|  |  |  |  |  |  |  |  |  |  |  |  |  |
| Gender |  |  |  |  |  |  |  |  |  |  |  |  |
| Male | 1,180 | + | 396 | 34 | + | 14 | 13 | + | 10 | 231 | + | 81 |
| Female | 1,090 | + | 435 | 29 | + | 12 | 14 | + | 9 | 211 | + | 96 |
|  |  |  |  |  |  |  |  |  |  |  |  |  |
| All | 1,129 | + | 419 | 31 | + | 13 | 14 | + | 9 | 220 | + | 90 |
