# Supplementary material for: Physical activity, energy requirements, and adequacy of dietary intakes of older persons in a rural Filipino community
Source: Nutr J. 2009 May 4;8:19. doi: 10.1186/1475-2891-8-19 (PMC2689250; doi:10.1186/1475-2891-8-19)
Supplement: Additional file 2 — Micronutrient intakes of the study participants, by age, gender and SES score. Intakes of iron, niacin, riboflavin, thiamin, calcium, vitamin A and vitamin C were not significantly different by age, sex or socio-economic status, except for vitamin A intake which was significantly higher for those with the highest SES. [file 1475-2891-8-19-S2.doc]

Additional file 2

Intakes of iron, niacin, riboflavin, thiamin, calcium, vitamin A and vitamin C were not significantly different by age, sex or socio-economic status, except for vitamin A intake which was significantly higher for those with the highest SES.

|  | **Iron (mg)** | **Niacin (mg)** | **Riboflavin (mg)** | **Thiamine (mg)** | **Calcium (mg)** | **Vitamin A (ug RE)** | **Vitamin C (mg)** |
| --- | --- | --- | --- | --- | --- | --- | --- |
| Age group |  |  |  |  |  |  |  |
| Near-old | 6.9 | 12.9 | 0.4 | 0.4 | 258.9 | 145.7 | 18.9 |
|  | 2.7 | 5.4 | 0.3 | 0.3 | 134.5 | 103.7 | 18.2 |
|  |  |  |  |  |  |  |  |
| Young-old | 6.7 | 10.6 | 0.4 | 0.4 | 241.7 | 144.0 | 18.5 |
|  | 3.0 | 5.2 | 0.2 | 0.2 | 149.7 | 72.7 | 16.8 |
|  |  |  |  |  |  |  |  |
| Old-old | 5.6 | 10.2 | 0.3 | 0.3 | 209.3 | 129.3 | 13.0 |
|  | 2.2 | 5.1 | 0.2 | 0.2 | 125.4 | 123.9 | 11.0 |
|  |  |  |  |  |  |  |  |
| Gender |  |  |  |  |  |  |  |
| Male | 7.0 | 12.4 | 0.4 | 0.4 | 260.8 | 138.1 | 17.1 |
|  | 2.5 | 5.4 | 0.2 | 0.2 | 162.7 | 92.9 | 12.2 |
|  |  |  |  |  |  |  |  |
| Female | 6.2 | 10.6 | 0.4 | 0.4 | 225.3 | 143.2 | 17.5 |
|  | 2.8 | 5.2 | 0.3 | 0.2 | 114.3 | 104.6 | 18.8 |
|  |  |  |  |  |  |  |  |
| SES score |  |  |  |  |  |  |  |
| 0 | 6.9 | 11.8 | 0.4 | 0.4 | 273.5 | 95.9 | 16.1 |
|  | 3.4 | 5.6 | 0.2 | 0.3 | 134.2 | 66.4 | 9.3 |
|  |  |  |  |  |  |  |  |
| 1 | 5.8 | 9.0 | 0.3 | 0.3 | 203.7 | 99.5 | 15.5 |
|  | 2.7 | 4.0 | 0.2 | 0.1 | 102.6 | 59.2 | 18.1 |
|  |  |  |  |  |  |  |  |
| 2 | 6.7 | 12.2 | 0.5 | 0.4 | 248.9 | 170.8 | 19.0 |
|  | 2.6 | 5.7 | 0.3 | 0.2 | 153.7 | 112.8 | 17.2 |
|  |  |  |  |  |  |  |  |
| All; Mean | 6.5 | 11.3 | 0.4 | 0.4 | 241.3 | 140.0 | 17.6 |
| All; SD | 2.8 | 5.4 | 0.3 | 0.2 | 139.6 | 100.7 | 16.4 |
